# Norm Values of Muscular Strength Across the Life Span in a Healthy Swiss Population: The COmPLETE Study

**DOI:** 10.1177/19417381221116345

**Published:** 2022-08-18

**Authors:** Eric Lichtenstein, Jonathan Wagner, Raphael Knaier, Denis Infanger, Ralf Roth, Timo Hinrichs, Arno Schmidt-Trucksaess, Oliver Faude

**Keywords:** ageing, health, life span, power, strength

## Abstract

**Background:**

Grip strength is used to estimate whole-body strength for health surveillance purposes. Explosive strength is considered important, yet economic measures able to detect early deterioration of neuromuscular capabilities are lacking. Whether handgrip maximum rate of force development (GRFD) or whole-body strength tests are better predictors of lower body power than handgrip maximum strength (GF_max_) and their trajectories throughout the life span are unknown.

**Hypothesis:**

GRFD should be more closely related to lower body power than GF_max_, and its trajectories over the life span should more closely follow that of lower body power.

**Study Design:**

Cross-sectional.

**Level of Evidence:**

Level 2b.

**Methods:**

A total of 613 healthy participants aged 20 to 91 years were tested for countermovement jump peak power, GF_max_, handgrip rate of force development, and midthigh pull peak force (MTP). Cubic splines and linear models were built for age trajectories, generalized additive models for quintile curves, and linear regression was used to assess predictive quality.

**Results:**

Peak power (P_max_) declined linearly to 60% of young adult level, with GRFD, GF_max_, and MTP remaining stable up to age 50 years and then declining more sharply to 52% to 71% of young adult levels. Trajectories were similar for male and female participants. GRFD (β = 0.17) and MTP (β = 0.08) were worse predictors of P_max_ than GF_max_ (β = 0.24) in models adjusted for age, sex, lean body mass, and vigorous physical activity levels.

**Conclusion:**

GRFD was not superior to maximum strength in predicting lower body power. For health surveillance purposes, it therefore appears that GF_max_ tests are more economical and equally good predictors of lower body explosive strength at older age. The data provided can be used as norm values for healthy subjects.

**Clinical Relevance:**

Incorporating countermovement jump testing for early detection of declines in explosive capabilities might be advised.

Muscular strength is an important aspect of physical health and its decline is associated with increased morbidity, disability, and mortality.^16,36,47^ A lack of strength predicts the inability to perform everyday tasks and thus a loss of autonomy.^40^ Lower strength is associated with reduced health-related quality of life (QoL) and functional status,^9,15,25,35^ but also cardiocirculatory and metabolic disorders.^10,24,36^ Strength declines with increasing age,^7,18,19,28,31,47,52^ with an accelerated loss reported after 60 years of age. An average reduction of maximum strength per decade of between 20% and 50% depending on age has been reported.^11^ The decline is caused by changes in muscle structure and neuromuscular innervation. Motor units are progressively lost, leading to an atrophy of the accompanying muscle fibers. Motor neuron firing rate, as well as their number, is reduced; fiber-type composition changes as fast-twitch muscle fiber decay becomes more pronounced, and contractile element molecular structure also changes.^20,38,54^ Importantly, some of those alterations might be independent of activity status.^34^ All those factors lead to a greater decrease in muscle function than in muscle mass, which has been used in the past as the most important aspect of sarcopenia. Accordingly, this has now been revised to include the loss of muscle function (dynapenia) as a symptom as well.^12^

For health surveillance purposes, handgrip dynamometry has been studied widely to assess strength as it is a simple, cheap, and reliable test for huge cohorts and has the potential to support diagnostic decisions in the clinical setting.^9,27,51^ It is promoted as a surrogate measure for whole-body strength,^5,8,37^ with leg strength being the most important aspect for maintenance of mobility and fall prevention.^23^ Yet, much of the association between leg and handgrip strength might be explained by age, sex, and anthropometric characteristics.^8,41^

When assessing strength, maximally produced force or torque, and explosive strength (ie, the rate at which force or torque can be produced) or power are frequently investigated. For the prevention of falls and prediction of real-life performance, the ability to produce force quickly is deemed more important than the maximum achievable force.^14,17,32^ Power capabilities are considered to decline faster than maximum strength, and training focusing on power and explosive execution of movement has shown more promising results than other types of strength training for the improvement of neuromuscular capabilities.^32^ Up to now, assessing explosive strength has required isokinetic devices or force plates, which makes it largely unsuitable for health surveillance purposes as those devices are not broadly available in the clinical or general practitioner setting, and require trained staff to conduct the assessments. Exploring the explosive capabilities of hand flexors could provide an insight into neuromuscular activation abilities, and economic alternatives could be developed to assess power ability in the clinical setting. To date, rate of force development data for handheld dynamometers have not been studied. Therefore, no information exists regarding the reliability of such measurements. Traditionally, assessing explosive strength has been shown to have lower reliability than maximum strength assessments.^1,44^ In addition, the validity of handgrip strength measurements for the estimation of leg strength and power has been investigated mostly with small sample sizes in laboratory studies with homogenous populations,^2,37^ making generalizability to a population for health surveillance purposes difficult.

So far, no study has investigated multiple strength parameters within the same population sample over ages covering the whole adult life span. For most of these measurements, reference values for healthy populations are also missing. Therefore, the aims of this study were to (1) establish norm values and age trajectories for a healthy population; (2) assess the ability to predict leg explosive strength from other strength measurements that do not require isokinetic devices, and can therefore be done in a clinical setting; and (3) establish whether the assessment of handgrip explosive strength has some additional value as compared with the assessment of maximal handgrip strength only.

## Methods

### Study Design and Participants

The COmPLETE health study is a cross-sectional investigation examining ageing trajectories of physical fitness components and health markers in a healthy population. Age brackets were defined as 20 to 29, 30 to 39, 40 to 49, 50 to 59, 60 to 69, 70 to 79, and 80+ years. Recruitment was designed in such a way that there was a similar number of participants in every age decade. All participants were recruited from around the area of the institution where the study was conducted. Unaddressed letters were sent to randomly chosen districts of the 11 neighborhoods around the institution’s location and 15 municipalities of an adjacent district. The latter represents a rural environment as compared with the urban environment of the institution’s location. Applicants were screened by telephone questionnaire and final eligibility was confirmed onsite by a physical examination by a medical doctor. To be included, participants had to be nonsmokers and have a body mass index (BMI) below 30 kg/m^2^. They were excluded from the study if they had manifest exercise-limiting chronic disease, were pregnant or breastfeeding, abused drugs or alcohol, had a blood pressure above 160/100 mm Hg, had Alzheimer’s disease or any other form of dementia, or were unable to follow the procedure of the study. Those criteria were chosen to attain a mostly healthy sample yielding reference data for a healthy population and to enable the detection of diversion in parameters from a healthy state (“health distance”).^3^

Participants were tested comprehensively for physical fitness and cardiovascular function and properties. This paper focuses on the analysis of the strength assessments conducted. Further details of the recruitment process, all measurements performed, and results of the primary outcomes can be found elsewhere.^26,48-50^ Participants were instructed not to drink any alcohol or exercise within 24 hours, not to drink beverages containing caffeine within 4 hours, and not to eat within2 hours before the appointment. About 30 minutes before the strength tests, participants were offered 500 mL plain water and an energy bar (170 kcal). The study was approved by the local ethics committee (approval number: EKNZ 2017-01451), funded by a governmental science foundation (grant no. 182815), registered at https://www.clinicaltrials.gov/, and was carried out in accordance with the latest version of the Declaration of Helsinki and the guidelines of good clinical practice. All participants gave written informed consent before participating.

### Procedures

Before physical fitness was tested, height, body mass, and body composition were measured. The order of the physical fitness tests was (1) gait analysis, (2) countermovement jump,(3) standing balance, (4) handgrip strength, (5) whole-body strength test, and (6) cardiopulmonary exercise testing. In addition, participants were instructed to wear a triaxial accelerometer (GeneActive Activinsights Ltd) on their wrist for 14 days to account for the confounding effect of a possibly reduced physical activity with increasing age.^49^ Accelerometer data were classified as light, moderate, or vigorous physical activity.^42^

### Strength Tests

The following tests were each performed 3 times with 1 minute of rest between successive trials.

Countermovement jumps were performed to assess the explosive strength of the legs and serve as the criterion for functional power capacities, also in the elderly.^29^ They were performed on a ground-reaction force plate (Leonardo Mechanograph, Novotec Medical). Participants were instructed to jump as high as possible. For those unable to jump, they were instructed to push as fast and hard as possible against the platform to generate maximum power. This way, the testing could be done without security concerns. Nevertheless, a study assistant stood behind participants to assist in the possible event of a fall. The peak power (P_max_ in W/kg) from the power-time graph normalized to lean body mass (LBM) was used for statistical analysis. Data were captured with a frequency of 500 Hz.

Handgrip strength was assessed using a handheld dynamometer (Leonardo Mechanograph GF, Novotec Medical). Participants stood upright holding the device with their elbows extended and were instructed to push as fast and hard as possible.^4^ Grip span was adapted to the individual hand size.^39^ From the force-time graph, the maximum value (maximum grip force [GF_max_ ] in N) and the steepest rise in force over a 150-ms window (handgrip maximum rate of force development [GRFD] in N/150 ms; first derivative of the force-time curve) are used as outcome parameters.^30^

A whole-body strength test was performed using an analog dynamometer (TTM, Muscular Meter). Participants were instructed to lift a bar attached by a chain to the platform they are standing on with maximum force while keeping their back straight.^43^ The test was performed at a knee angle of 110° and mimics the midthigh pull (MTP) test common in athletic settings.^6^ The instruction was to pull as hard as possible. On the analog dynamometer, which is attached to the pulling chain, the maximum achieved force is displayed in kilograms. The achieved pulling force (kg) normalized to LBM was used for statistical analysis.

For all measurements, the best of 3 trials was used in the calculation, except for the reliability analysis, where all successful trials were used and only participants with 3 valid attempts (tasks executed as instructed) were included.

### Statistical Analysis

Participant characteristics and outcomes for every decade are presented with means and SDs. To assess the trajectories over the life span of the different outcome parameters, all individual datapoints were normalized to the youngest age brackets mean for the respective sex. Cubic smoothing splines with 5 knots were constructed for the visualization of the trajectories and linear models for the decline in performance relative to the 20 to 29 year age bracket were calculated for both sexes. The estimator for the influence of age on performance with 95% CIs are compared. Separate regression models were built for the 20 to 39, 40 to 64, and 65 years and older groups to explore whether the decline is stronger at older age. We chose 65 years as this is frequently the age at which most people retire. For visualization of norm values, age- and sex-specific quantile curves were calculated using generalized additive models for location, scale, and shape (GAMLSS, R package version 5.3-4).^45^ The age trajectories were modeled using P-splines. We adopted the Bayesian information criterion to select the conditional distribution that offered the best compromise between model complexity and goodness of fit.

To assess the predictive quality of strength parameters for lower limb explosive power, linear models were constructed with P_max_ as the dependent variable and age, sex, LBM, physical activity, and respective strength parameters as independent variables. For the selection of the physical activity parameters, linear regression models were built including sex; age; and light, moderate, and vigorous physical activity and the parameter with the strongest association to P_max_ was selected. In addition, this analysis was separately conducted for younger (20-39 years), middle aged (40-64 years), and older (65+ years) subjects. Standardized b coefficients and model strength (*R*^2^) were compared between the investigated parameters. Differences in b coefficients of at least 0.1 can be considered small but potentially relevant.^22^

As a supplementary analysis, we calculated test-retest reliability using the spreadsheets provided.^21^ The typical error in percentage (coefficient of variation) was used as reliability statistic alongside the intraclass correlation coefficient (3,1), each together with 95% CIs. This analysis was also carried out for the 3 previously mentioned age categories.

## Results

### Age Trajectories and Norm Values

**Figure 1.**
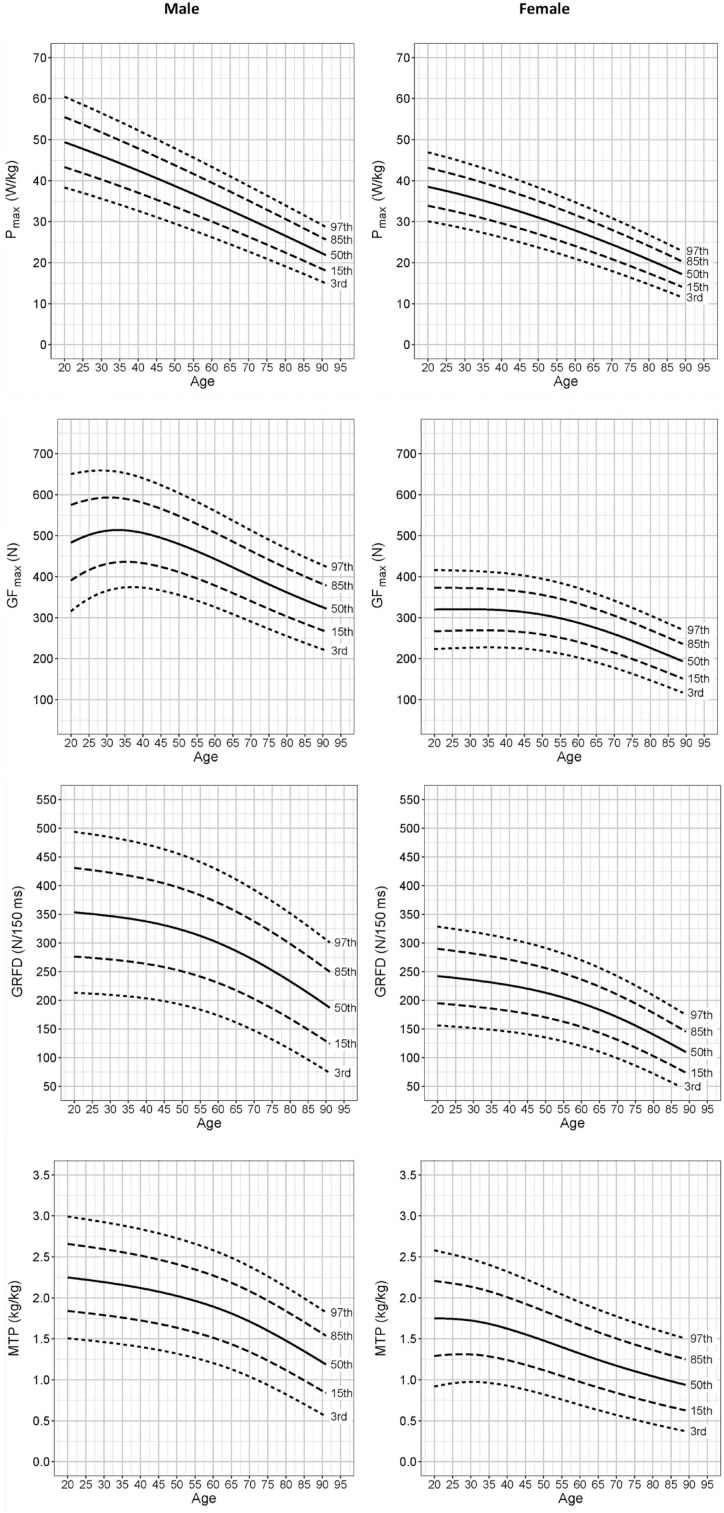
Percentiles for strength parameters by age and sex. GF_max_, handgrip maximum strength; GRFD, handgrip maximum rate of force development; MTP, midthigh pull peak force; P_max_, countermovement jump peak power adjusted to body weight.

The number of tested participants across all age ranges was 613, with 88 to 96 participants per decade, except for the oldest age bracket (Table 1). The average performance in the 4 strength parameters for every age bracket is displayed in Table 1 along with participant characteristics. Table 2 shows the strength parameter values normalized to the performance of the youngest brackets (Table 2). The norm value quantiles for this data for men and women are presented in Figure 1.

**Table 1.** Participants characteristics and strength performance parameters

Sex	Bracket, years	N	Age, years	BMI, kg/m^2^	LBM, kg	P_max_, W/kg	MTP, kg/kg	GF_max_, N	GRFD, N/s
Male	20-29	47	25.0 (2.7)	23.4 (2.4)	65.0 (8.3)	56.0 (6.6)	2.57 (0.38)	491 (87)	336 (82)
	30-39	47	34.5 (3.0)	24.1 (2.2)	64.8 (7.3)	54.2 (6.2)	2.61 (0.54)	520 (77)	344 (65)
	40-49	41	45.3 (3.1)	24.1 (2.4)	63.6 (6.6)	48.3 (7.3)	2.50 (0.42)	481 (78)	341 (68)
	50-59	48	55.0 (3.0)	25.0 (2.4)	63.9 (7.2)	46.8 (6.1)	2.44 (0.41)	473 (53)	320 (69)
	60-59	47	63.8 (2.6)	24.4 (2.3)	59.9 (5.9)	42.4 (5.6)	2.43 (0.41)	440 (54)	290 (69)
	70-79	46	73.6 (2.5)	24.8 (2.5)	56.4 (5.2)	38.8 (10.0)	2.14 (0.44)	377 (65)	250 (60)
	80+	36	83.3 (3.0)	25.5 (2.7)	55.0 (5.9)	34.0 (5.7)	1.82 (0.45)	342 (60)	220 (74)
Female	20-29	42	24.9 (2.4)	21.9 (3.0)	46.6 (6.6)	49.2 (5.3)	2.20 (0.45)	316 (53)	234 (50)
	30-39	41	34.0 (3.2)	22.2 (2.8)	47.4 (4.2)	46.4 (5.0)	2.35 (0.40)	322 (46)	229 (39)
	40-49	50	44.9 (3.2)	22.6 (2.5)	49.3 (5.3)	43.8 (4.7)	2.01 (0.45)	321 (49)	229 (42)
	50-59	46	54.3 (2.7)	22.8 (2.1)	46.3 (5.2)	40.0 (4.7)	1.96 (0.46)	295 (46)	203 (41)
	60-59	48	64.8 (3.0)	23.1 (2.6)	42.4 (4.4)	38.4 (6.4)	1.68 (0.44)	274 (45)	182 (37)
	70-79	42	73.5 (2.4)	23.5 (3.0)	42.3 (3.9)	32.6 (3.9)	1.71 (0.41)	249 (47)	161 (44)
	80+	31	83.9 (2.4)	23.3 (2.7)	40.0 (4.6)	29.2 (5.7)	1.41 (0.33)	210 (44)	123 (35)

Values are means and SDs. BMI, body mass index; GF_max_, maximum grip force; GRFD, handgrip maximum rate of force development; LBM, lean body mass; MTP, midthigh pull peak force; P_max_, countermovement jump maximum power.

**Table 2.** Age bracket performance standardized to youngest bracket in percent values

Sex	Bracket, years	P_max_	MTP	GF_max_	GRFD
Male	20-29	100 (12)	100 (15)	100 (18)	100 (24)
	30-39	97 (11)	102 (21)	106 (16)	103 (20)
	40-49	86 (13)	97 (16)	98 (16)	101 (20)
	50-59	84 (11)	95 (16)	96 (11)	95 (21)
	60-69	76 (10)	94 (16)	90 (11)	86 (21)
	70-79	69 (18)	83 (17)	77 (13)	74 (18)
	80+	62 (10)	71 (18)	70 (12)	66 (22)
Female	20-29	100 (11)	100 (20)	100 (17)	100 (21)
	30-39	94 (10)	107 (18)	102 (14)	98 (17)
	40-49	89 (10)	91 (20)	102 (16)	98 (18)
	50-59	91 (10)	89 (21)	93 (15)	87 (18)
	60-69	78 (13)	76 (20)	87 (14)	78 (16)
	70-79	66 (8)	78 (19)	79 (15)	69 (19)
	80+	60 (12)	64 (15)	67 (14)	53 (15)

Values are means and SDs. GF_max_, maximum grip force; GRFD, handgrip maximum rate of force development; MTP, midthigh pull peak force; P_max_, countermovement jump maximum power.

**Figure 2.**
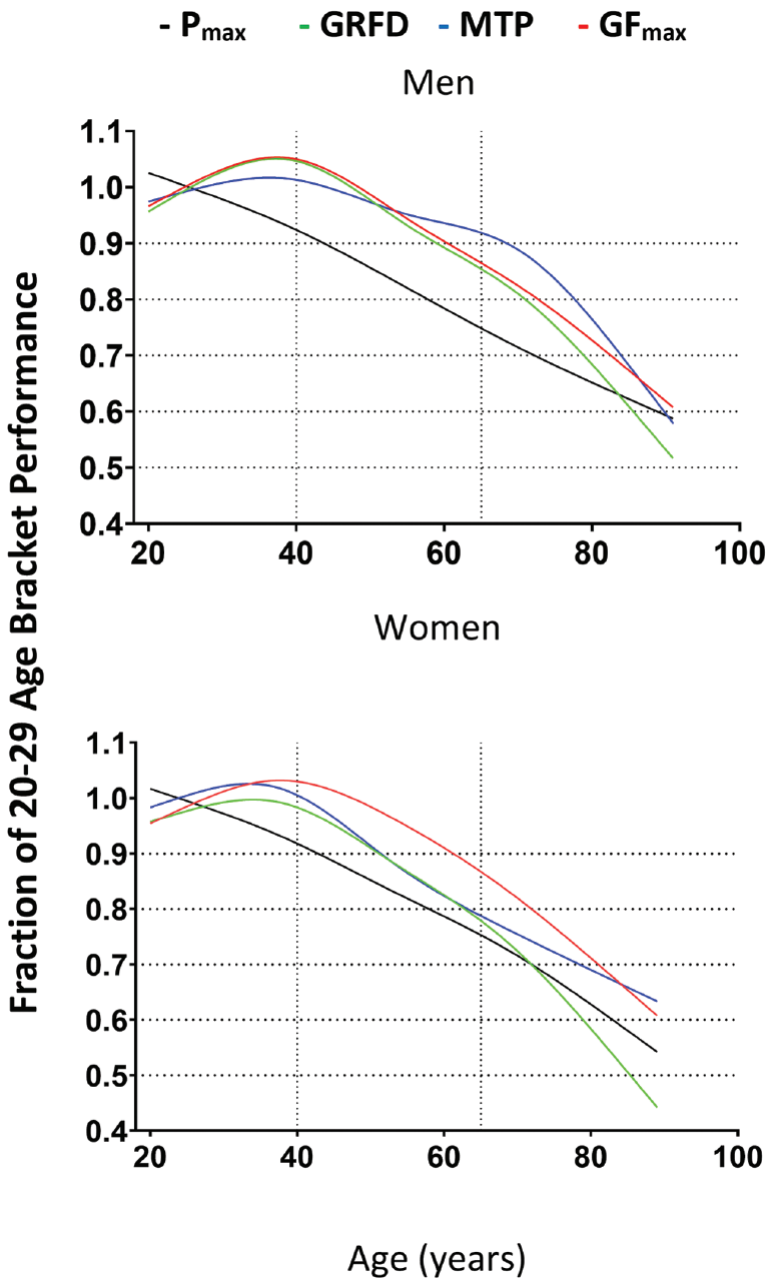
Cubic smoothing spline with 5 knots for the 4 different strength parameters across the age span. GF_max_, handgrip maximum strength; GRFD, hand grip maximum rate of force development; MTP, midthigh pull peak force; P_max_, countermovement jump peak power adjusted to body weight.

The oldest men retained on average 62%, 71%, 70%, and 66% of the countermovement jump peak power, GF_max_, GRFD, and MTP, respectively. Women in the oldest bracket on average performed at 60%, 64%, 67%, and 53% for those parameters. The decline in jumping peak power could be observed from the beginning of the investigated age range (Figure 2). However, performance for handgrip strength parameters and MTP strength remained stable throughout the 5th decade of life (Figure 2). Starting at age 40 years, the percentage decline for all 3 parameters was very similar. Considering the observed trajectories, projected performance in GRFD at the latest stages of life seems to decline even more strongly than leg power, especially for woman, where this can be observed starting at age 70 years.

### Performance Decline

The estimated decline in performance per decade from the linear models can be found in Table 3. Of note, GRFD is estimated to decline more strongly than the other parameters between the ages of 40 and 65 years, but a similar decline is also compatible with most of the data. The decline was, on average, stronger for the 65+ bracket than the 40 to 64 year bracket, except for P_max_ for men. The strongest decline per decade was observed for GRFD in women over 65 years of age (-13.8%) and for MTP in men over 65 years of age (-13.0%). The least loss of performance at older age was observed for women’s MTP over 65 (-5.7%) and men’s P_max_ over 65 (-5.3%). The youngest bracket showed a decline in P_max_, while the other strength parameters remained stable or increased with age in this population.

**Table 3.** Estimated decline per decade in percentage with 95% CIs

Sex	Bracket	P_max_	MTP	GF_max_	GRFD
Female	20-39	−4.8 [−9.1; −0.5]	+8.5 [+0.8; 16.2]	+6.1 [−0.1; +12.3]	+0.4 [−7.3; +8.2]
	40-64	−7.8 [−10.4; −5.3]	−5.2 [−10.6; −0.2]	−4.5 [−8.2; −0.7]	−8.6 [−13.0; −4.1]
	65+	−10.0 [−13.2; −6.7]	−5.7 [−11.0; −0.3]	−9.7 [−14.1; −5.4]	−13.8 [−18.7; −8.8]
Male	20-39	−3.0 [−7.3; +1.2]	+4.7 [−2.1; +11.4]	+6.5 [+0.4; +12.6]	+3.3 [−4.8; -+11.3]
	40-64	−5.6 [−8.5; −2.7]	−1.8 [−6.0; +2.4]	−5.6 [−8.7; −2.5]	−9.4 [−14.5; −4.3]
	65+	−5.3 [−9.7; −1.0]	−13.0 [−18.0; −8.0]	−9.9 [−13.7; −6.0]	−12.4 [−18.2; −6.7]

GF_max_, handgrip maximum force; GRFD, handgrip peak rate of force development; P_max_, countermovement jump peak power; MTP, midthigh pull peak force.

### Regression Results

The prediction of P_max_ from potential field tests including age, sex, LBM, and vigorous physical activity revealed only very small associations between MTP and P_max_ across all subgroups with standardized estimates ranging from 0.08 to 0.11. Comparatively, maximum grip strength was a better predictor, with standardized estimates between 0.24 and 0.37, except for the 40 to 64 age range (β = 0.09). GRFD was a similarly good predictor in the youngest group (β = 0.34), but less so for the oldest (β = 0.08) and the middle-aged (β = 0.15) groups (Table 4). The strength of the models declined with increasing age groups for all parameters. Of the variation in P_max_, 59% to 60% can be explained by either strength parameter model, reducing to 20% to 30% within the subgroups as age remains the primary predictor of P_max_. There was no relevant difference in model strength between parameters.

**Table 4.** Standardized regression estimates (β) for prediction of P_max_ from MTP, GF_max_, and GRFD for different subgroups and model strengths (adjusted *R*^2^)^*a*^

	MTP			GF_max_			GRFD		
Age	β	*P*	*R* ^2^	β	*P*	*R* ^2^	β	*P*	*R* ^2^
20-100	0.08	0.017	0.59	0.24	<.001	0.60	0.17	<.001	0.59
20-40	0.11	0.11	0.32	0.37	0.004	0.34	0.34	<.001	0.37
40-64	0.10	0.18	0.28	0.09	0.45	0.28	0.15	0.083	0.28
65-100	0.08	0.32	0.20	0.29	0.02	0.22	0.08	0.42	0.20

GF_max_, handgrip maximum force; GRFD, handgrip peak rate of force development; LBN, lean body mass; MTP, midthigh pull peak force; P_max_, countermovement jump peak power.

a All models are adjusted for age, sex, LBM, and vigorous physical activity.

## Discussion

In this study, trajectories of 4 different strength parameters across the life span in a healthy population ranging from 20 to 91 years of age were investigated. We compared new and economic strength tests for potential health surveillance purposes with widely studied tests and parameters. The results of our study show that trajectories over the life span in these parameters were similar in people older than 40 years of age, but that lower body explosive power declined nearly linearly throughout the observed age range. We observed an accelerated decline with increasing age in all other parameters. The data provided herein can be used to compare clients and patients with healthy peer cohorts to assess their muscular fitness level. Similar declines in strength were observed for men and women. The assessment of handgrip-derived explosive power or MTP strength did not seem to be a better predictor for leg power than the established maximum grip strength in any subgroup. Based on the data gathered, additional assessment of these new parameters in this field does not justify the increased resources needed. Handgrip maximum strength remains an economic, reliable, and valid tool for health surveillance. To assess the effectiveness of interventions, using lower body power tests remains important, as only 60% of the variability in the lower body power data can be explained by our model incorporating age, sex, LBM, physical activity, and GF_max_ (Table 4).

Previous studies have reported different rates of decline in strength and power depending on investigated region (legs or hands), muscle contraction type (isometric, isokinetic), test type (closed or open chain), and sex.^13,18,28,31,33,46^ For example, maximum strength of the dorsiflexor muscles has been reported to decrease by 1% to 2% per year, and the power of the same muscles by 3.5% per year in men.^33^ These data are larger than the declines in our study (0.5% to 1% decrease per year in P_max_ and 1% per year in GF_max_ beyond the age of 65 years) and also cover a large range of decline due to the small number of subjects (39) with corresponding greater uncertainty in the data. Arguably, investigating nonloadbearing musculature and musculature not used for forward propulsion during locomotion, might lead to reporting of stronger declines due to lack of use of these muscles in activities of daily living (ADL). Also, we investigated subjects at every age, whereas, for example, McNeil and colleagues extrapolated declines from young (26.1 years), old (64.6 years), and very old (83.3 years) groups to the whole male adult life span. Studies investigating the decline in lower body peak power in closed chain exercises (like the countermovement jump or chair rise test) report a decrease of around 50% between ages 20 and 68 to 80.^13,46^ However, the oldest age bracket in our study retained around at least 60% of the youngest bracket’s performance. Possibly, the rigorous inclusion of only healthy participants led to this discrepancy, while comorbidities not controlled for could confound decreases with age in other studies. Most notably, inclusion criteria considering BMI and smoking status were not used by the aforementioned studies.^13,46^

In regard to handgrip strength, trajectories and declines in this study are consistent with previous studies, including the steadiness of GF_max_ throughout the 3rd, 4th, and partly the 5th decade of life.^18,28,31^ Against the initial assumptions, there was no discernible difference in trajectories of GF_max_ and GRFD and, apparently, we were not able to gain additional insights into neuromuscular activation abilities. This might be explained by the fiber-type composition of the forearm being skewed toward slow-twitch muscle fibers, as the main purposes of the hand in ADL lie in tasks of fine coordination rather than powerful tasks.

In regression models adjusted for age, sex, LBM, and vigorous physical activity, the variance in the criterion variable P_max_ was best explained by GF_max_ (β = 0.24) followed by GRFD (β = 0.17) and MTP (β = 0.08) (Table 4). We expected the MTP to show a stronger relationship with P_max_ than grip strength parameters, as both are closed chain exercises requiring the whole body for force production. From our data, it might be assumed that 2 different constructs are measured and that the MTP is not a more suitable surrogate for P_max_ in the general population than maximum grip force. In athletic populations, moderate-to-strong correlations between MTP and countermovement jump performance or clean performance have been found, albeit unadjusted for the possible confounders we included in our analysis.^6,53^ In fact, disregarding adjustment in our data would also show moderate correlations between P_max_ and MTP (*r* = 0.475, *P* < 0.001). Alas, the correlations between P_max_ and handgrip strength parameters would be stronger (*r* = 0.55 for GF_max_ and GRFD). Despite the preferential economy of the MTP measurement, tests of maximum handgrip strength, which might be even more economical, might be most suitable for health surveillance purposes. One interesting finding is the lack of a relationship between GF_max_ and P_max_ between the ages of 40 and 64 years, where GRFD was a slightly better predictor of P_max_ although both relationships were rather small (β = 0.09 and 0.15, respectively). Potentially, for the early detection of reduced whole-body neuromuscular capacities, handgrip strength measurements might not be suitable and closed chain lower body explosive tests, like the countermovement jump, should be recommended. At old age, handgrip strength tests might be sufficient, as results of the latter are more closely associated to countermovement jump power. Those assessments are likely more sensitive than reductions in the performance of ADL or QoL, which likely follow as a result of already substantially declined strength. In fact, when controlling for age, sex, LBM, and vigorous physical activity, gait speed was not correlated with P_max_ (β = 0.075) or QoL (SF8) (β < 0.01) in our healthy sample.

We saw good reliability in the conducted tests with the exception of handgrip rate of force development (Supplementary Material). This is in line with the previously reported differences in reliability between maximum force and explosive force.^1,44^

The data provided herein could be used to assess the neuromuscular age of a patient and differences from healthy peers could be calculated. This might help give insights into preclinical transitions from a health to an unhealthy state.^50^ From the data presented, it seems that a performance of around 60% of the young adult level can be considered sufficient to maintain autonomy at old age, and diversions from this level of performance might be indicative of an increasingly unhealthy state. Furthermore, when conducting interventions aiming to improve strength parameters, this data can be used to assess the magnitude of intervention effects by considering the results of the reliability analysis (Supplementary Material).

## Limitations

Despite the huge age range, the investigated population can be considered very homogenous as they are all fairly active and healthy. As shown in the Appendix, available in the online version of this article, there was little variance in activity levels between the ages. Generalizing these findings to the whole population, where various health threatening conditions are very prevalent and inactivity is present in the majority of the population, should therefore be done with caution. Relationships between the investigated parameters are potentially much stronger if a more diverse population were to be investigated. Due to the tested population, no cutoff values for potentially clinically relevant reduced neuromuscular performance can be provided, but these do exist in abundance for GF_max_.^40,51^ Of note, the 2 oldest age groups (80+) performed, on average, close to or below the cutoff values of approximately 35 kg for men and 20 kg for women, despite being active and healthy individuals. All other age groups performed well over these cut-offs, providing further proof that the investigated population can be considered healthy from a clinical standpoint. Establishing cutoff values for P_max_, especially in middle age to discern healthy and unhealthy strength levels should potentially be done in the future.

## Conclusion

Lower body power declines linearly from young adulthood to old age in healthy people. At age 80 years, around 60% of young adult maximum is still achieved. Surrogate measurements like GF_max_ can predict this decline. The prediction is less accurate before retirement age as trajectories of the surrogate parameters are relatively stable up to the age of 50 years followed by an accelerated decline after the age of around 65. Using MTP or GRFD likely provide no improvement to predicting lower body strength compared with established tests. The data presented can be considered norm values for healthy populations over the whole life span.

## Supplemental Material

sj-docx-1-sph-10.1177_19417381221116345 – Supplemental material for Norm Values of Muscular Strength Across the Life Span in a Healthy Swiss Population: The COmPLETE Study Supplemental material, sj-docx-1-sph-10.1177_19417381221116345 for Norm Values of Muscular Strength Across the Life Span in a Healthy Swiss Population: The COmPLETE Study by Eric Lichtenstein, Jonathan Wagner, Raphael Knaier, Denis Infanger, Ralf Roth, Timo Hinrichs, Arno Schmidt-Trucksaess and Oliver Faude in Sports Health: A Multidisciplinary Approach
