# Supplementary material for: Norm Values of Muscular Strength Across the Life Span in a Healthy Swiss Population: The COmPLETE Study
Source: Sports Health. 2022 Aug 18;15(4):547–57. doi: 10.1177/19417381221116345 (PMC10293570; doi:10.1177/19417381221116345)
Supplement: sj-docx-1-sph-10.1177_19417381221116345 – Supplemental material for Norm Values of Muscular Strength Across the Life Span in a Healthy Swiss Population: The COmPLETE Study [file sj-docx-1-sph-10.1177_19417381221116345.docx]

**APPENDIX: Supplementary Material**

1. **Reliability**

The reliability analysis revealed over the whole sample small coefficients of variation for MTP (95 % Confidence Interval: 4.1 – 4.4 %), P_max_ (2.5 – 2.7 %) and F_max_ (2.9 – 3.2 %) values. For the GRFD a coefficient of variation of 8.8 to 9.5 % was observed indicating higher variability in the performance between trials for this method. ICCs of 0.97 to 1.0 were observed for all parameters. The good reliability of MTP, P_max_ and F_max_ was mostly independent of age, except slightly increased CV for MTP in the 40-64 subgroup (6.3 – 7.2 %) and P_max_ in the 65+ subgroup (3.7 – 4.3 %). The greater variability between tests for the GRFD parameter was similar for all age groups. The reliability statistics for the three age groups can be found in Table A1.

| Table A1. Intra-session test-retest reliability statistics. Values are 95 % confidence intervals. | | | | | | |
| --- | --- | --- | --- | --- | --- | --- |
|  | | | | | | |
|  | 20-39 | | 40-64 | | 65+ | |
|  | CV | ICC | CV | ICC | CV | ICC |
| P_max_ (W/kg) | 1.6; 1.9 | 0.99; 1.00 | 1.4; 1.6 | 1.00; 1.00 | 3.7; 4.3 | 0.95; 0.97 |
| MTP (kg) | 1.4; 1.6 | 1.00; 1.00 | 6.3; 7.2 | 0.99; 1.00 | 1.8; 2.1 | 1.00; 1.00 |
| GF_max_ (F) | 3.2; 3.7 | 0.98; 0.99 | 2.7; 3.1 | 0.99; 0.99 | 2.8; 3.2 | 0.99; 0.99 |
| GRFD (N/s) | 8.9; 10.4 | 0.94; 0.96 | 7.2; 8.1 | 0.96; 0.97 | 9.5; 10.9 | 0.96; 0.97 |
|  | | | | | | |
| CV: coefficient of variation (%), ICC: intraclass correlation coefficient; P_max_: countermovement jump peak power, MTP: mid-thigh pull peak force, GF_max_: hand grip maximum force; GRFD: hand grip peak rate of force development. | | | | | | |

The investigated tests can all be considered reliable, but hand grip RFD seems less reliable than the other investigated tests. Potential reasons for that might be, that performing explosive hand grip tasks is not usually required in daily activities and subjects therefore had problems performing this task reliably.

1. **Physical Activity**

In the whole sample, only vigorous physical activity was predictive of P_max_ when adjusted for age and sex (model 1). Interestingly, in the 65+ age group none of the physical activity parameters was a good predictor of P_max_ (model 2; Table A2).

| Table A2: Regression models for the prediction of P_max_ from physical activity parameters. | | | | |
| --- | --- | --- | --- | --- |
|  | | | | |
| Parameters | Model 1 | | Model 2 | |
|  | β | p | β | p |
| Intercept | 0 | < .001 | 0 | < .001 |
| Sex (0 = male, 1 = female) | -0.66 | < .001 | -0.60 | < .001 |
| Age | -0.713 | < .001 | -0.37 | < .001 |
| LPA | 0.01 | 0.878 | -0.06 | 0.464 |
| MPA | 0.02 | 0.586 | -0.01 | 0.957 |
| VPA | -0.07 | 0.023 | -0.05 | 0.487 |
|  |  |  |  |  |
| Model 1: whole sample, Model 2: only including subjects 65 years or older, β: standardized estimate, p: p-value, LPA: light physical activity, MPA: moderate physical activity, VPA: vigorous physical activity, P_max_: counter movement jump peak power. | | | | |
